# Towards Effective Consensus Scoring in Structure-Based Virtual Screening

**DOI:** 10.1007/s12539-022-00546-8

**Published:** 2022-12-23

**Authors:** Do Nhat Phuong, Darren R. Flower, Subhagata Chattopadhyay, Amit K. Chattopadhyay

**Affiliations:** 1 Department of Mathematics, College of Engineering and Physical Sciences, Aston University, Birmingham, B4 7ET UK; 2 Life and Health Sciences, Aston University, Birmingham, B4 7ET UK; 3 Acculi Labs Pvt. Ltd., Bangalore, Karnataka 560098 India

**Keywords:** Molecular docking, Machine learning, Consensus scoring, Virtual screening

## Abstract

Virtual screening (VS) is a computational strategy that uses in silico automated protein docking *inter alia* to rank potential ligands, or by extension rank protein–ligand pairs, identifying potential drug candidates. Most docking methods use preferred sets of physicochemical descriptors (PCDs) to model the interactions between host and guest molecules. Thus, conventional VS is often data-specific, method-dependent and with demonstrably differing utility in identifying candidate drugs. This study proposes four universality classes of novel consensus scoring (CS) algorithms that combine docking scores, derived from ten docking programs (ADFR, DOCK, Gemdock, Ledock, PLANTS, PSOVina, QuickVina2, Smina, Autodock Vina and VinaXB), using decoys from the DUD-E repository (http://dude.docking.org/) against 29 MRSA-oriented targets to create a general VS formulation that can identify active ligands for any suitable protein target. Our results demonstrate that CS provides improved ligand–protein docking fidelity when compared to individual docking platforms. This approach requires only a small number of docking combinations and can serve as a viable and parsimonious alternative to more computationally expensive docking approaches. Predictions from our CS algorithm are compared against independent machine learning evaluations using the same docking data, complementing the CS outcomes. Our method is a reliable approach for identifying protein targets and high-affinity ligands that can be tested as high-probability candidates for drug repositioning.

**Graphical Abstract:**

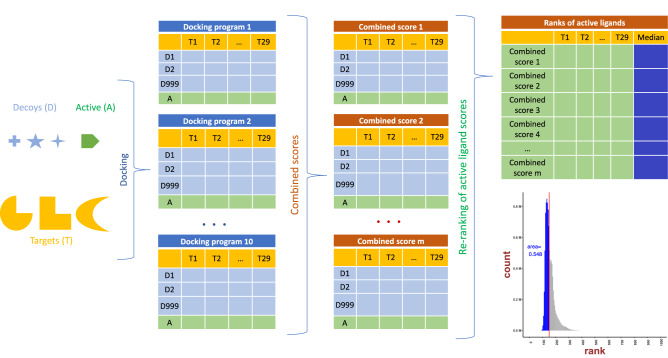

## Introduction

Apart from being time- and resource intensive, the success rate of traditional drug discovery is low [1, 2]. Drug Repurposing (DR), the evaluation of approved or safety-evaluated drugs as treatments for new or different diseases, has mostly relied on haphazard, trial-and-error drug discovery to match prospective drug candidates to cognate target proteins [2, 3]. Next-generation DR methods involve computationally intensive automated screening of extant compounds against protein or nucleic-acid targets [4]. This method has come to be known as Virtual Screening (VS). Virtual Screening (VS) protocols can computationally map compound libraries against biological targets to detect compounds with potential biological activities while eliminating unsuitable compounds [5–7]. Such in silico virtual screening can assess large numbers of compounds rapidly, including molecules yet to be synthesized.

Docking is a widely used computational method to predict the likelihood of meaningful complementarity between small molecule compounds and protein targets [8, 9]. Despite major advances in algorithms and hardware, the quality of discrimination available within current docking programs remains sub-optimal [10]. When we combine thousands of proteins with tens of thousands of ligands, the task becomes computationally challenging. To surmount this obstacle, efforts have been made to combine docking programs to derive consensus scores.

A major advance in VS began with the implementation of screening combining inputs from multiple VS platforms, a methodology popularly known as “consensus scoring” (CS) [11, 12]. Trial-and-error implementations of consensus CS generates superior ligand–protein matching when compared to individual VS [11–13]. Initially conceptualized by Charifson [14], consensus scoring algorithms have been employed in both structure-based and ligand-based virtual screening [15, 16] and are now becoming the norm [17], making contributions to the identification of drug candidates for Ebola [18] and Zika [19]. Recently, Scardino et al [20] have employed a new consensus method that uses ranking and pose of the docked ligands to ensure more robust virtual screening. A key advantage of consensus scoring over individual VS is its ability to reduce false positives and negatives in virtual screening [14], thereby optimizing the time and resources required.

Consensus scoring protocols rely on established statistical (e.g. skewness-kurtosis, regression) measures [11, 12], complemented by machine learning [21–23]. The prerequisite for statistical consensus scores is a homologous set of initial scores. For instance, the docking scores can be uniformly generated [13] or rescored with the same docking engine [14]. For heterogeneous docking scores spanning a range of docking programs with varying units and ranges, the individual scores are first normalized using either rank transform [11, 12], minimum–maximum scaling [15] or *z*-score scaling [24] before the combination, which can contribute to data loss.

The present study makes use of a different normalization procedure that ensures convergence without data loss by using a three-tier approach. Tier 1 involves docking data from the enhanced DUD-E repository (http://dude.docking.org/) (1000 ligands docked against 29 MRSA-oriented targets) using ten popular and easily accessible (open access) docking programs: ADFR, DOCK6, Gemdock, Ledock, PLANTS, PSOV-ina, QuickVina2, Smina, Autodock Vina and VinaXB. The choice is governed by reported individual success rates, e.g. DOCK6 at 73.3% [25], Autodock Vina at 80% [26], Gemdock at 79% [27], ADFR at 74% [28], Ledock at 75% [29], PLANTS at 72% [30], PSOVina 63% [31], QuickVina2 63% [32], Smina more than 90% [33] and VinaXB 46% [34]. The docking programs were randomly chosen focusing only on the need to use an open-sourced architecture that could be utilized on a terminal-based (that is, without a Graphical User Interface) Linux/Unix frontend, a requirement of the Midlands Supercomputing Cluster (now named SULIS) that we used for computations. Tier 2 combines data from all 10 scores using statistical (linear and nonlinear) models belonging to four universality classes. Tier 3 normalizes VS data from Tier 2 through a novel calibration of the individual best score (Smina in our case) against the respective probability density functions (PDF). Since PDF data is non-dimensional, normalization is guaranteed and is without meaningful information loss.

This study also outlines a self-consistent mechanism of understanding how multiple docking combinations ensure better convergence, answering questions relating to a possible improvement in CS accuracy with additional docking entries. The study convincingly demonstrates that a finite number of docking programs are required for the highest available accuracy. The precise number required may vary depending on the specific choice of docking programs used.

We analyze the strength of our novel CS model against Methicilin Resistance *Staphylococcus aureus* (MRSA). The bacterium is a prime example of antimicrobial resistance, accounting for up to 12% of hospital infections between 2011 and 2014 in the UK [35]; 323,700 infected patients in 2017 incurring an approximate cost of $1.7 billion [36]. In this work, we focus on MRSA essential genes as de facto targets for potential repurposed drugs acting as anti-MRSA antibiotics, arguing that inhibiting any essential gene should impair the biological activity of the whole bacteria. Benchmark is done using MRSA targets comparing different MRSA protein structures to targets obtained from the Directory of Useful Decoys—Enhanced (DUD-E).

## Methods

### Target and Ligand Selection

DUD-E decoys and active ligands are docked to MRSA structures that are structurally similar to their DUD-E targets. The idea is to evaluate the veracity of the docking structure used without the decoys necessarily binding to the targets, as in Graves, et al. [37] 351 essential genes from the Database of Essential Genes [38] are aligned with PDB structures using BLAST [39], resulting in 113 target structures identified in the Protein Data Bank (PDB) [40]. To benchmark MRSA-oriented targets effectively, instead of re-docking DUD-E ligands against their respective targets, we compare protein structures of MRSA proteins and DUD-D targets. 102 target protein structures from DUD-E [41] are structurally aligned with those of 113 MRSA proteins using the Dali server [42] and visual inspection. 29 pairs of structurally similar MRSA—DUD-E are recorded. For each DUD-E set of decoys and active ligands after filtering with Lipinski Rule of Five [43] for drug-like compounds, 999 decoys and one active ligand are reserved for each target.

We docked 1000 DUD-E ligands initially against 1 (DUD-E or MRSA) target. This is what we see in Table 1, the last column. While the initial docking involved DUD-E ligands against DUD-targets, we later substituted DUD-targets with structurally similar MRSA targets, individually and collectively. For example, the MRSA target 4DQ1 is reasonably similar in structure to the DUD-E target TYSY, or (3WQT, 5JIC) are similar to HXK4 and could be substituted.

**Table 1:** List of structurally similar DUD-E and MRSA targets

DUD-E targets	DEF	DYR	ADA, ALDR GLCM, PYRD	DHI1, INHA	HXK4	TYSY	
MRSA target	1LM4	2W9H	3M9Y, 3T05	3OSU, 4D44	3WQT, 5JIC	4DQ1	
			4HB7, 4TO8, 5BOE				

Targets in the same column share similar structures using results from the Dali server. 999 decoys and one active ligand DUD-E ligands were docked against MRSA targets that shared similar structures instead of their DUD-D targets

### Molecular Docking

Ten docking programs were chosen due to their ease of use and prominence as follows: ADFR [28], UCSF DOCK [29], Gemdock [27], Ledock [29], PLANTS [30], PSOVina [31], QuickVina2 [32], Smina [33], Autodock Vina [26] and VinaXB [34]. All protein structures used were downloaded from the Protein Data Bank (PDB) [40]. Prior to docking, protein structures have water and ions removed and are then protonated. Decoys and ligands are prepared similarly. Binding site prediction is carried out using FTSite server [43] for DOCK, Gemdock, Ledock, PLANTS, PSOVina, QuickVina2, Smina, Autodock Vina and VinaXB while ADFR uses its own package Autosite [45]. 999 decoys and 1 active ligand are docked against all 29 MRSA targets. Each docking program generates various ligand conformations and orientations within a binding pocket (pose) and uses its underlying scoring function to estimate the likelihood of binding for each pose. The best scoring pose is retained for each decoy and ligand.

### Normalization

To compare with other consensus scores, common methods of normalization are applied to docking scores before combination. We employed the three commonly-used normalization procedures. (A) Ranking: Ranks represent docking scores for each target assigned against ascending ranks. This implies that ligands with more negative scores rank higher. (B) Minimum–maximum Scale (referred to hereafter as min–max scale). Scores for each target are rescaled to a [0; 1] domain and then subtracted from the minimum score. The result is then divided by the difference between the maximum and the minimum score. (C) *z*-score. The min–max docking scores are mean averaged or zero-centered and rescaled. A drawback of these normalization methods is that they shift the relative distribution of scores, which may cause a loss of information.

### Consensus Algorithms

Molecular docking is a process that generates different conformations of poses of ligands and predicts the intermolecular interactions using sets of physicochemical properties, including hydrogen bonding and hydrophobicity. Consensus scoring creates an overall score consistent with the ensemble representation of the 3D molecule rather than an individual pose. To avoid information loss while using normalization, our consensus algorithms combine information from all docking programs and then generate the following four independent optimized functional ensemble data representations:

1a $$S_{\text{c}} = \mathop \sum \limits_{i = 1}^{10} \mathop \sum \limits_{j = 0}^{20} x_{i,j} S_{i,j}^n$$

1b $$S_{\text{c}} = \mathop \sum \limits_{i = 1}^{10} \mathop \sum \limits_{j = 0}^{20} x_{i,j} {\text{abs}}\left[ {S_{i,j}^n } \right]$$

1c $$S_{\text{c}} = \mathop \sum \limits_{i = 1}^{10} \mathop \sum \limits_{j = 0}^{20} x_{i,j} \left( {S_{i,j}^n - \overline{S_i }} \right)^n$$

1d $$S_{\text{c}} = \mathop \sum \limits_{i = 1}^{10} \mathop \sum \limits_{j = 0}^{20} x_{i,j} {\text{abs}}\left[ {\left( {S_{i,j}^n - \overline{S_i }} \right)^n } \right]$$

Here *S*_c_ is the combined score. *S*_*i*_ is the docking score of ligands for programs *i* = 1, 2, …, 10. *x*_*i*_ are coefficients of the docking programs *i* (ADFR, DOCK, Gemdock, Ledock, PLANTS, PSOVina, QuickVina2, Smina, Autodock Vina and VinaXB); these are the weights for docking outcomes. *S* is the mean of the set from program *i*. *n* represents the combinatorial order, real values only (*n* = 1 implies linear combination). Equations (1a–1d) were iterated over a total of 20 [9] ensembles involving 10 docking programs, each weighing between 0 and 1, incremented in steps of 0.05 each. *S*_*i*_ represents the arithmetic means of the docking scores of all ligands for the same target for each docking program used. The rank of active ligands before and after combination was compared to evaluate the improvement produced by our consensus algorithm.

### Consensus Outcomes

The mean or median rank of active ligands can be used to compare the performance of consensus scores and individual docking programs. Here, we use the median rank of active ligands across all targets, which provides a better threshold than mean ranks. We dock active ligands and rank them with the medians as thresholds across all 29 targets. The median rank of active ligands is expressed as the recovery rate of virtual screening performance: when 50% of active ligands are retrieved at a certain proportion of the ligand library. The fraction of the library screened is defined as the arithmetic mean of the median rank over 1000 ligands.

We compared the result against other consensus scores: Mean (MEAN), Median (MED), Minimum (MIN), Maximum (MAX), Euclidean Distance (EUC), Cubic Mean (CBM), Exponential Consensus Rank (ECR) [46] and Deprecated Sum Rank (DSR) [47] across ten sets of normalized docking scores (*S*_*i*_) as follows:

2a $${\text{MEAN}} = {\text{mean}}\left\{ {S_1 ,S_2 ,S_3 , \ldots , S_{10} } \right\}$$

2b $${\text{MED}} = {\text{median}}\left\{ {S_1 ,S_2 ,S_3 , \ldots , S_{10} } \right\}$$

2c $${\text{MIN}} = {\text{minimum}}\left\{ {S_1 ,S_2 ,S_3 , \ldots , S_{10} } \right\}$$

2d $${\text{MAX}} = {\text{maximum}}\left\{ {S_1 ,S_2 ,S_3 , \ldots , S_{10} } \right\}$$

2e $${\text{EUC}} = \left[ {\mathop \sum \limits_{i = 1}^{10} S_i^2 } \right]^{1/2}$$

2f $${\text{CBM}} = \left[ {\mathop \sum \limits_{i = 1}^{10} S_i^3 } \right]^{1/3}$$

2g $${\text{ECR}} = \mathop \sum \limits_{i = 1}^{10} \exp \left( {S_i } \right)$$

2h $${\text{DSR}} = \frac{{\sum_{i = 1}^{10} S_i }}{{{\text{maximum}}\left\{ {S_i } \right\}}}$$

Models defined through Eq. (2g) and (2h) assume the rank of the scores, not the scores themselves. Model from Eq. (2h) is without the maximum of the list.

## Results and Discussion

29 targets were obtained from the DUD-E repository. For each target, 999 decoys and 1 active ligand were randomly chosen. These 1000 ligands were then docked against each target using ten docking programs (ADFR, DOCK, Gemdock, Ledock, PLANTS, PSOVina, QuickVina2, Autodock Vina and VinaXB), producing 10 matrices of 1000 × 29 (active ligands are intentionally located at the 1000th row). For consensus scores, the docking results of each ligand-target pair were combined using Eqs. (1a–1d). While anal***yzing a new set of combined scores, for each target, all combined scores were picked in descending order, starting with the best binding energy. The medians of these re-positioned values were then used to calculate the histogram leading to the probability distribution function.

### Statistical Ranking of Docking Scores (DUD-E Database)

In this study, we used the median ranking order for evaluation. First, active ligands for 29 targets were randomly chosen and then ranked across a 1000 ligand (docked) arrays. A random selection leads to a median rank of 500. The median ranks obtained from 10 docking programs verified that the median ranks of active ligands (250 from ADFR) were better than those obtained from a random selection, as detailed in Table 2.

**Table 2:** Performance of docking programs across 29 targets

	ADFR	DOCK	Gemdock	Ledock	PLANTS	PSOVina	QuickVina2	Smina	Autodock Vina	VinaXB
Target 1	761	344	712	235	446	900	838	641	637	613
Target 2	32	77	166	38	203	67	171	150	77	125
Target 3	337	826	330	514	83	685	83	62	191	224
Target 4	22	95	77	78	2	530	159	38	77	193
Target 5	769	46	137	385	178	375	332	190	242	388
Target 6	2	103	192	392	17	119	11	1	1	1
Target 7	110	445	32	98	667	388	635	497	475	521
Target 8	776	635	941	637	416	940	907	980	930	797
Target 9	334	571	331	490	94	376	250	194	231	260
Target 10	210	93	123	83	28	709	44	48	53	59
Target 11	339	64	523	387	146	376	367	299	390	374
Target 12	255	82	89	694	14	112	15	125	6	7
Target 13	861	831	316	806	646	418	696	423	438	211
Target 14	302	123	174	71	593	607	569	568	498	563
Target 15	881	523	758	843	362	837	823	922	931	877
Target 16	57	112	57	59	196	230	106	103	90	140
Target 17	275	477	666	276	169	27	143	101	139	166
Target 18	892	837	79	176	21	236	47	6	51	73
Target 19	446	669	264	312	295	338	487	383	305	338
Target 20	58	2	20	67	31	8	51	21	16	22
Target 21	688	731	456	422	360	442	406	294	583	457
Target 22	542	14	43	122	5	93	94	16	104	105
Target 23	168	123	9	403	13	194	342	227	365	387
Target 24	44	423	203	611	163	80	29	38	44	44
Target 25	842	795	84	448	382	287	157	260	185	240
Target 26	723	992	453	336	62	442	245	89	150	357
Target 27	408	41	619	782	74	17	185	43	622	100
Target 28	173	909	261	943	35	251	7	2	7	1
Target 29	646	831	138	545	422	527	636	664	476	669
Median	337	423	192	387	163	375	185	150	191	224

Each number represents the rank of 29 separate active ligands ranked against a set of 1000 ligands after docking to their targets. Best functioning docking programs that are capable of clearly distinguishing active ligands and decoys are identified by ranks close to 1. The median value represents the average performance of each docking program across all 29 targets

Compared against the statistical scores defined in Eqs. (2a–2h), our rank-based normalization consistently returned low scores, complementing the predictions from the consensus algorithm. Table 3 tabulates the consensus scores against varying normalization.

**Table 3:** Average performance of traditional consensus scores across various normalization

	Mean	Median	Min	Max	EUC	CBM	ECR	DSR
Min–max normalization	228	246.5	184	202.5	206	201	217	224
Rank normalization	191	195	271	205.5	176	174	207.5	183
*z*-score normalization	203	209	256	231	1000	220	191	205

**Fig. 1:**
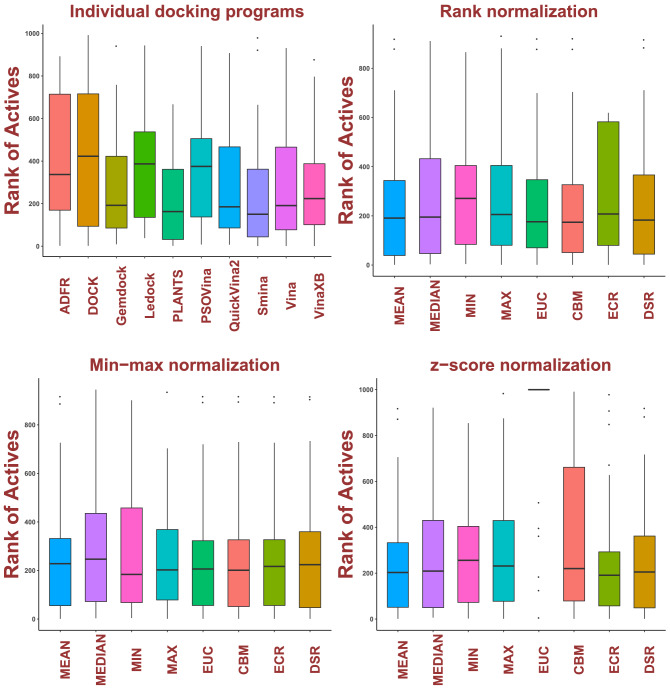
Box plot of ranks from programs and consensus scores (From left to right: ADFR, DOCK, Gemdock, Ledock, PLANTS, Vina, scored as in Eqs. (2a–2h). The lines parallel to the *x*-axis in each box represent the median

**Fig. 2:**
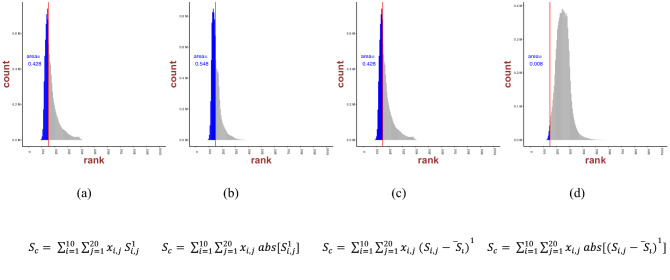
Consensus scores, defined as area fraction (to the left of the best-performing individual docking score marked with a straight line) of the total histogram area, evaluated for linear regression, i.e. *n* = 1 as in Eqs. (1a–1d)

After docking and calculating the ranks of active ligands across 29 targets, Smina returned the lowest median rank of 150, followed by PLANTS with median ranks of 163 and 185 in QuickVina2. Autodock Vina and Gemdock show comparative median ranks of 191 and 192. Surprisingly, the highly popular DOCK generated the worst score (median rank of 423). In general, Autodock Vina show promising results. Based on this evaluation, Smina was the single best-performing docking program for the DUD-E ligands. Converted to recovery rate, the percentage median scores of the docked results are 33.7%, 42.3%, 19.2%, 38.7%, 16.3%, 37.5%, 18.5%, 15%, 19.2% and 22.4% for ADFR, DOCK, Gemdock, Ledock, PLANTS, PSOVina, QuickVina2, Smina, Autodock Vina and VinaXB, respectively. See Fig. 1. The boxplot for Smina shows the ratio of the box height from the median to 0 (median marked by the black line) divided by 1000 is 15%. Thus, if we take 15% of the best-ranked ligands for Smina, we have half of the active ligands. Substituting the median baseline with mean and mode did not change the outcome. The first plot of Fig. 2 shows the individual performance of docking programs while the three other plots illustrate the conventional consensus scores from ten docking programs after normalized with various normalization methods.

As demonstrated in Fig. 1, these conventional consensus scores show no noticeable improvement compared to individual docking programs, given the choice of normalization methods.

### Novel Consensus Scores

For each docking program, the median ranks of active ligands across 29 targets have been used and plotted using histograms. To establish the improved performance of consensus scores (CS) over individual docking, we compared scores from the individual best performer Smina against the CS score. This was estimated from the leftward areas (since binding energy is negative) of our best-performing individual docking platform (Smina, identified by the solid line close to the maxima of the histograms). Greater the area, the better the CS score (compared to Smina).

**Fig. 3:**
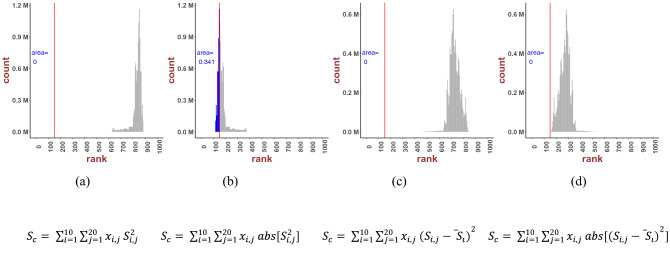
Consensus scores, defined as area fraction (to the left of the best performing individual docking score marked with a straight line) of the total histogram area, evaluated for *n* = 2 as in Eqs. (1a–1d)

As clearly demonstrated in Fig. 3, the linear consensus model was consistently the best performer, with the CS docking score progressively declining with increasing values of *n*. We found that three out of the four linear combinations (*n* = 1) demonstrated higher ranks compared to the individual best performer Smina [82, 83 and 82 for model (1a–1c), respectively]. Another trend was the dominance of the odd n values against their even counterpart. This was to be expected, as the docking scores were energies, hence negative. This could be compensated for by the absolute (consensus) values [as in models in Eq. (1b) and Eq. (1d)]. Model (1d) was the worst scorer, while linear combinations of models (1a–1c) showed similar behavior with approximate best ranks and comparable histograms (non-normalized probability density functions).

As evident from Figs. 2 and 3, linear regression (Figs. 2) over the set of 10 docking scores involving our ligand–protein sets returned better docking score than nonlinear regression (Figs. 3). Results for higher-ordered consensus regression are provided in the Appendix.

Area ratio is the area of the histogram of median ranks obtained from novel consensus models that show better ranking than that of the best individual docking program. Rank improvement is defined as the increment of rank compared to that of the best program.

### Consensus Model Accuracy Convergence

**Fig. 4:**
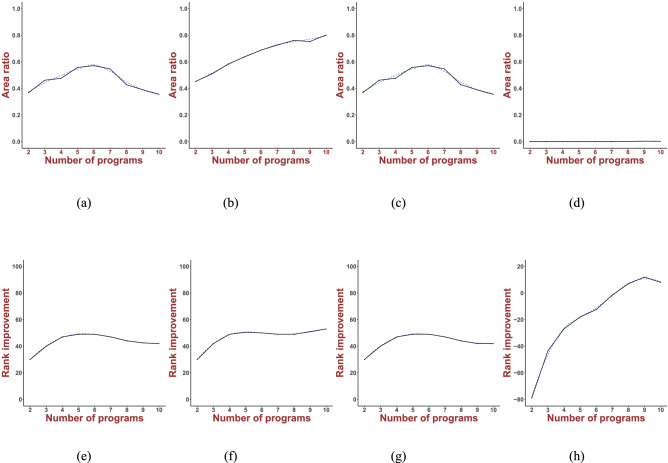
Rank improvement versus the number of docking programs. From left to right column: area ratio of model (1a–1d); upper figures: area ratio versus the number of docking programs; lower figures: rank improvement versus the number of docking programs

To evaluate the strength of linear combination in each model, we estimated the correlation between the number of docking programs and the consensus performance. Two following types of measures were calculated: area ratio and rank improvement, relative comparisons of which are shown in Table 4. The model in Eq. (1a) defines an explicit correlation between the number of docking programs and the consensus outcome. The area ratio increased from 2 to 7 programs and then became saturated after approximately 8 docking combinations (Fig. 4b). Similarly, rank improvement drastically increased from 2 to 4 programs and flattened after 5 programs (Fig. 4f). A comparison between these two measures suggested that having large numbers of docking programs does not necessarily enhance overall performance. Models (1a) and (1c) showed similar saturation patterns both for area ratio and rank improvement. The consensus effect increases monotonically with combinations of two programs, reaching a maximum value after 5 or 6 programs (Fig. 4a, c, e, g). Model (1d) showed poor improvement in both area ratio and rank, with the area ratio mostly remaining zero (Fig. 4d) while rank showed negative changes around *n* = 8 programs (Fig. 4h), indicating no improvement.

**Table 4:** Performance of novel consensus scores

Power	$S_{\text{c}} = \mathop \sum \limits_{i = 1}^{10} \mathop \sum \limits_{j = 1}^{20} x_{i,j} S_{i,j}^n$		$S_{\text{c}} = \mathop \sum \limits_{i = 1}^{10} \mathop \sum \limits_{j = 1}^{20} x_{i,j} {\text{abs}}\left[ {S_{i,j}^n } \right]$		$S_{\text{c}} = \mathop \sum \limits_{i = 1}^{10} \mathop \sum \limits_{j = 1}^{20} x_{i,j} \left( {S_{i,j} - \overline{S_i }} \right)^n$		$S_{\text{c}} = \mathop \sum \limits_{i = 1}^{10} \mathop \sum \limits_{j = 1}^{20} x_{i,j} {\text{abs}}\left[ {\left( {S_{i,j} - \overline{S_i }} \right)^n } \right]$	
	Best rank	Area ratio	Best rank	Area ratio	Best rank	Area ratio	Best rank	Area ratio
1	82	0.532	83	0.648	82	0.532	119	0.020
2	558	0	109	0.541	395	0	152	0
3	109	0.450	109	0.413	112	0.107	177	0
4	579	0	109	0.289	399	0	174	0
5	110	0.295	110	0.180	118	0.085	177	0
6	572	0	111	0.117	399	0	17	0
7	111	0.137	111	0.078	116	0.086	177	0
8	556	0	112	0.047	399	0	182	0
9	112	0.070	112	0.038	119	0.087	179	0
10	543	0	112	0.005	399	0	179	0

A possible reason for the lack of convergence in Fig. 4b, f is the use of absolute values, causing gradual increments (‘accumulation’ effect) as the number of docking programs increases, unlike in models (1a) and (1c) for which the consensus accuracy converges faster by 4 or 5 programs.

**Fig. 5:**
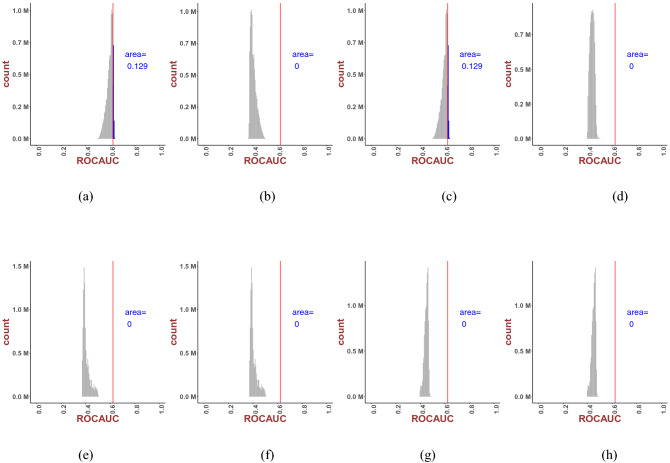
Histogram of consensus models using ROC for evaluation: From left to right column: area ratio of model (1a–1d) respectively; upper figures: power 1; lower figures: power 2. The area to the right of the red line represents a better ROC after combination than the ROC of Smina (0.623)

To compare our novel rank-based CS algorithm with more conventional statistical algorithms, such as the Receiver Operating Characteristic (ROC), we evaluated histograms of consensus models (DUD-E data) (Fig. 5) using CS scoring of the ROC data. The consensus results showed only minor improvement in the ROC area when compared to Smina. We found that conventional statistical approaches such as enrichment factor did not highlight the advantage of the CS method, unlike the previous (Figs. 2, 3) rank-based method.

Here, we used small incremental changes to the relative weights and compared each against the other, retaining only the top-scoring ones. The quality of this prediction compares favorably with results from machine learning, as shown below. Table 5 converges to a ranking of the top DUD-E ligand candidates based on CS scoring.

**Table 5:**
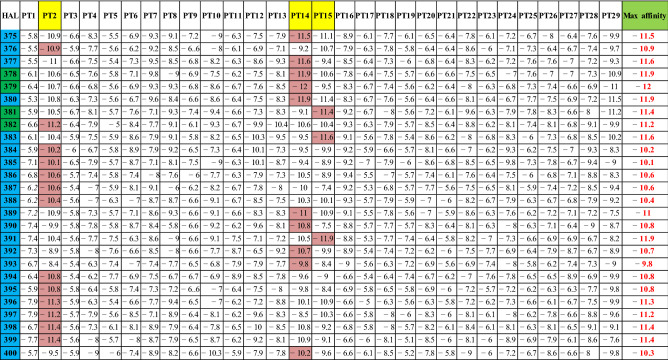
Mapping HALs to the corresponding PPTs—‘Reverse Modeling’

### Complementary Machine Learning Evaluation

High-Affinity Ligands (HAL)-Prime Protein Target (PPT) (“High-Affinity-Ligand–Protein-Complex” or HPCs hereafter) are identified using *k*-Means Clustering (*k*-MC). See Table 6. The HPCs are ‘reverse mapped’ to the original active database using mutual “affinity scores” between the 40 HALs and 29 PPTs for each dataset. From the 400 HAL-TPC datasets, three sets of test data (26 each) were chosen for evaluation. The first is set ‘A’, comprising the *last* 26 rows (ligands 375–400) of the original dataset. The second test set, set ‘B’, comprises the *middle* 26 rows (ligands 251–276). The third test set is set ‘C’ and comprises the *first* 26 rows (ligands 1–26) of the original dataset. The test data was chosen to indicate the HPCs of the original dataset. The observations are shown below: observation-1: PPT identification, observation-2: HAL identification and observation-3: HPC identification. A summary observation describes the outcome of the complementary ML model.

**Table 6:**
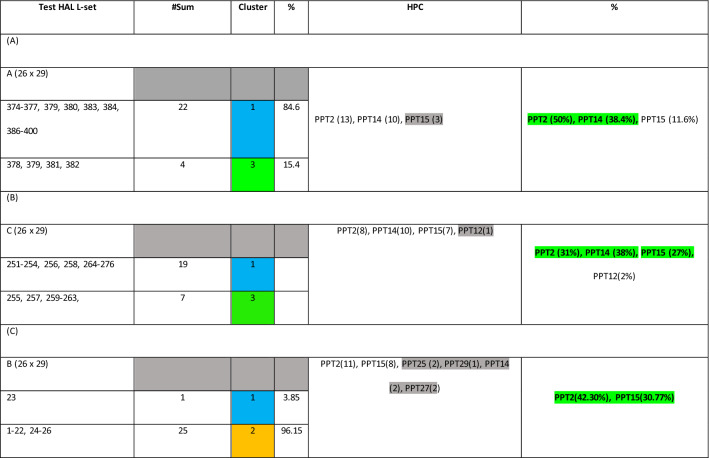
Evaluation of relationships among HAL test data ‘A’, ‘B’, ‘C’ and PPTs based on clusters

#### Observation 1: Prime Protein Target (PPT) Identification

From k-MC, three distinct high-quality clusters were obtained. Using Euclidean distance measures across all datasets around the centroids of each cluster, Clusters 1, 2 and 3 are found to contain 62%, 19% and 18% of the ligands, respectively. This information has been reversed mapped to indicate which ligands have high affinity to the protein targets (see Table 6a–c). k-MC identifies **PPT2**, **PPT14** and **PPT27** as the prime protein targets (see Table 5).

#### Observation 2: High Affinity Ligand (HAL) Identification

From the test sets, observed by reverse mapping, it can be noticed that in Test set ‘A’: ligand numbers 379, 380, 381 and 392 (15%) have a maximum affinity towards PPT 14. Test set ‘B’: ligand numbers 259, 260, 261 (11%) have a maximum affinity towards PPT 14. Test set ‘C’: ligand numbers 12, 14 and 17 (11%) have a maximum affinity towards PPT27, PPT27 and PPT2, respectively.

#### Observation 3: HPC Identification

- PPT14 ⟷ HAL #259–261, #379–381, #392
- PPT27 ⟷ HAL #12, #14
- PPT2 ⟷ HAL #17

The Machine Learning (ML) protocols used to identify the 14th protein target as a good match against ligands 259–261, 379–381 and 392, respectively, followed by the 27th protein target matching ligands 12 and 14, and finally the 2nd protein target finding a good match with ligand number 17. These are the top drug candidates identified within the ML landscape that offers an independent assessment of possibilities. Note, this is not to suggest that any approach, e.g. consensus is necessarily better or inferior to the other, e.g. ML. While not within the scope of this study, we are considering stage-wise comparison of both predictions, consensus and ML, versus molecular dynamics predictions that should provide insight into the stability of the proposed drug candidates.

#### Summary Observation (Table 6)

Therefore, from 72 Test ligands, 14% are found to be HALs, whereas out of 29 Protein targets, 3 PPTs (10%) are HPCs. These HPCs can be proposed as candidates for experimental analysis and subsequent drug design. The method used can only explore the important HPCs numerically and is not suitable for ranking, which requires in vitro experiments and empirical evaluation of individual HPCs.

Based on these experiments, we conclude that PPT2 (average HPC is 41.1%) is the highest-ranked protein candidate, as most HALs show high affinity towards it, followed by PPT14 (average 25.46%), and then PPT15 (average 23.12%).

#### Reverse Mapping (Table 6)

**Fig. 6:**
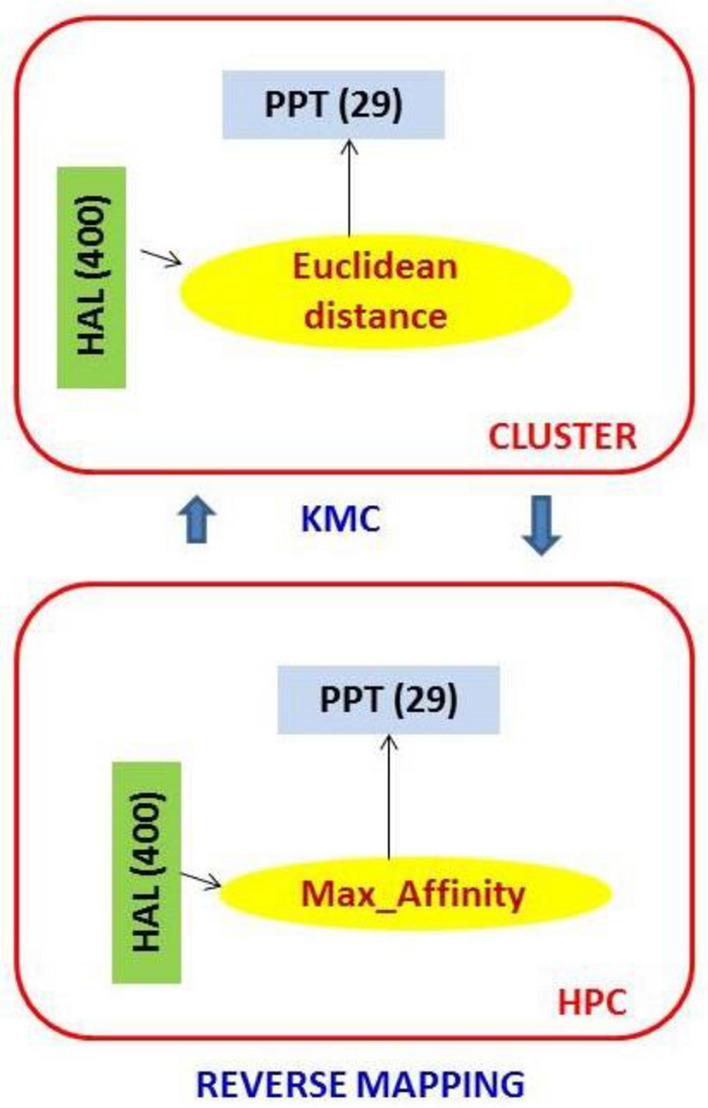
The ML evaluation technique using KMC and reverse mapping

In this table, ‘HALs’, ‘PPTs’**, and their respective ‘Affinity scores’ are ‘green’, ‘yellow’, and ‘magenta’ colored boxes. Table 6 also shows HPCs obtained from test data ‘B’ and ‘C’ similarly. Figure 6 explains our clustering-to-reverse-mapping approach to HAL-PPT affinity evaluation.

## Conclusions

We investigated consensus scoring algorithms using MRSA datasets and ten docking programs (ADFR, DOCK, Gemdock, Ledock, PLANTS, PSOVina, QuickVina2, Smina, Autodock Vina and VinaXB). Our performance benchmark was the median rank of active ligands. We also compared the individual docking programs with conventional consensus scores (minimum, maximum, mean, median, reciprocal rank and Euclidean distance). We also included the newly reported Exponential Consensus Rank score [45].

Prior to consensus scoring, we altered the distribution of docking scores with 12 pre-normalization (with molecular weight and number of heavy atoms) and normalization (rank, min–max scaling, and *z*-scores) thresholds to offer a direct comparison with commonly used statistical consensus scores. Comparisons indicate that our dataset is not sensitive to conventional consensus scores, showing no improved rank compared to 150 in Smina. Nonetheless, our novel consensus scores consistently perform better than individual docking programs on the MRSA benchmark dataset. In this work, we used raw docking scores from ten docking programs (ADFR, DOCK, Gemdock, Ledock, PLANTS, PSOVina, QuickVina2, Smina, Autodock Vina and VinaXB). Due to the exhaustive search of possible combinations, there was no requirement for data normalization. Results suggest that our model gives better rankings of active ligands across this benchmark dataset.

A key outcome is the preponderance of linear combinations of docking scores showing improved active ligand ranking over non-linear consensus approaches. Given that such complex systems are known to be inherently nonlinear, such linear mapping is interesting and potentially more useful than nonlinear scores. In Eqs. (1a–1d), odd-ordered combinations show consistently better performance than their even-ordered counterparts. Our findings also indicate that linear combinations using absolute values (model 1b) converge towards a better functional relationship linking the number of docking programs and consensus performance. While consensus prediction accuracy is proportional to the increasing number of docking programs (see Fig. 4), it is not a monotonically diverging quantity. Rather, it saturates beyond a finite number of combinations, typically 5–7 for our sets of ligands and MRSA proteins. This is a remarkable feature of the consensus approach. It should allow for the systematic substitution of weaker docking programs with programs exhibiting a higher scoring accuracy, as they arise over time since consensus scoring will always outperform even the best-performing individual docking program.

Both as a benchmarking exercise and from the perspective of complementing extant consensus predictions, we used machine learning (k-means clustering) to identify the prime protein targets (PPTs) and high-affinity ligands (HALs). While CS offers a probabilistic list of ideal combinatorial candidates between the given ligand and protein sets, clustering methods can identify the principal PPTs and HALs. This is a key outcome of this study, as we can now suggest a self-consistent algorithm capable of finding the correct MRSA drug candidates suitable for wet lab experiments.

The combination of CS and ML offers a straightforward approach able to combine docking scores from diverse docking platforms with higher overall efficiency than any individual docking program (CS) and predict PPTS and HALs (ML). This model can also be used in ligand-based virtual screening, where normalization usually requires data fusion. We will expand our study to include a greater range of docking programs as well as targets other than MRSA. We also plan to explore other descriptors, such as negative and/or fractional statistics. Our algorithm can lead to repositioned drug candidates while simultaneously offering a complementary prediction platform based on machine learning. We note that machine learning and our algorithm are complementary protocols; they should not be expected to benchmark any strategy, but rather assist in identifying overlap in prediction.

## Data Availability

Protein and ligand data from the open-sourced repository (http://dude.docking.org) have been used. Data modelling codes are all ours, based on a combination of Matlab_R2020a, R4.1.1 and python3.8, which are proprietary only. Executable codes could be available on request.
